# A CT study of the femoral and sciatic nerve periacetabular moving in different hip positions

**DOI:** 10.1186/s13018-020-01937-7

**Published:** 2020-09-11

**Authors:** Yagmur Isin, Onur Hapa, Yavuz Selim Kara, Ali Ihsan Kilic, Ali Balcı

**Affiliations:** 1 Department of Orthopedic Surgery, Kurtalan State Hospital, 56500 Siirt, Turkey; 2 Department of Orthopedic Surgery, Dokuz Eylül University, 35320 İzmir, Turkey; 3 Department of Radiology, Dokuz Eylül University, 35320 İzmir, Turkey

**Keywords:** Neurovascular injury, Hip, Periacetabular osteotomy, Sciatic nerve location

## Abstract

**Background:**

Femoral and sciatic nerves could be damaged during various stages of the periacetabular osteotomy. Changing the position of the hip could be the most effective way of preventing nerve injuries. The purpose of the present study was to investigate the distances of the nerves to various bony landmarks with different hip positions in computerized pelvic scanograms of healthy adults.

**Materials and methods:**

Fifteen healthy male adults (30 hips) (age 30 ± 6) were included. Scans were performed at three different hip positions measured by goniometer (neutral “N,” flexion (30–45°) + abduction (30–45°) + external rotation (20°) “F” and neutral+ abduction (30–45°) + external rotation (20°) (N_abext_) at three different levels (sourcil “1,” the middle of the femoral head “2,” and lower border of triradiate cartilage “3.”

**Results:**

At the sourcil level, the femoral nerve was found to be the furthest distance from the anterior acetabulum in the neutral position compared to flexion or neutral plus abduction, external rotation (*p* < 0.001). For the sciatic nerve, at level 2, hip flexion resulted in a greater distance than in the neutral position (*p* = 0.001). For level 3, hip flexion caused a decrease in the distance of the sciatic nerve to the acetabulum compared to both neutral positions (N or N_abex_) (*p* = 0.001).

**Conclusions:**

During a pubic cut of the osteotomy, the femoral nerve moves closer to the anterior acetabulum wall with hip flexion or abduction plus external rotation. During an ischial cut, the sciatic nerve gets closer to the ischium with hip flexion while it moves away from the bone during retroacetabular cut.

Level-III Study

## Introduction

Bernese periacetabular osteotomy (PAO) is the main treatment choice for residual acetabular dysplasia [1]. In classical PAO that described by Ganz, Smith-Peterson approach is used [1]. The first nerve that must be protected is the lateral femoral cutaneous nerve. By lateral incision of the tensor fascia lata, the lateral cutaneous nerve can be protected. After exposing bone surfaces, there are 3 main bone cuts. The first one is the ischial cut, and during this cut, the hip is placed in 45° of flexion and is slightly adducted [1]. The sciatic nerve is under the risk of damage, and to protect the sciatic nerve, care should be taken not to drive the chisel too deeply through the lateral cortex, especially with the hip flexed and adducted [1]. The second cut is the pubic cut, and for this cut, the hip should be slightly flexed and adducted and the iliopsoas and femoral neurovascular structures should be protected with a retractor that is driven into the pubic ramus. The third cut, ilium cut, consists of two cuts (supraacetabular and retroacetabular). The supraacetabular cut is performed leg extended and slightly abducted for the outside cut and slightly flexed for the inside cut [1]. The hip is once again flexed and adducted to relax the medial soft tissues for the retroacetabular cut [2].

However, it is not without complications since the main nerves of the hip (femoral nerve and sciatic nerve) are at risk. The reported prevalence of main nerve injuries could be as high as 15% (prevent of nerve in Sierra) which could be devastating for the patient [3]. There is uncertainty about the recovery time and whether it will be full or partial recovery [2]. Depending on this, various precautions have been prescribed to prevent nerve injuries including the positioning of the leg during osteotomy cuts, EMG monitoring, careful usage of retractors, usage of special osteotomes, and v.s [2, 4].

Among these, adjusting the position of the leg during bone cuts remains a controversial topic. Conflicting recommendations exist concerning the position of the hip during the 1st step, “ischial cut,” of the procedure. Most recommend bringing the hip to an extension during the first cut, which will relax the sciatic nerve and take it further away from the ischium [2, 4]. However, one recent magnetic resonance (MR) study reported that the hip flexion, abduction, and external rotation resulted in an increase in the distance between the nerve and ischium compared to a neutral position or pure flexion [5].

For the femoral nerve, excessive lateral tilt and medial displacement of the proximal fragment may lead to kinking in the nerve at the fragment edge, especially at full extension of the hip. Thus, it is recommended to flex the hip to decrease the tension at the nerve [2, 4].

A few studies exist regarding the position of the femoral nerve to bony landmarks. One computed tomography (CT) study reported that the femoral neurovascular bundle gets closer to the acetabular wall as it comes inferiorly along the anterior wall and recommended placing retractors as high as possible; anterior inferior iliac spine being the safest (neurovas proximity) [6]. Another study reported a decrease in the length of the nerve (decrease of stretch) with hip flexion, abduction, and external rotation compared to hip extension. However, no study has described the distance of femoral neurovasculature to the bone at various hip positions [7].

The purpose of the present study was to investigate the distances of the nerves to various bony landmarks at different hip positions (neutral, neutral+abduction+external rotation, flexion+abduction+external rotation) in computerized pelvic scanograms of healthy adults. The hypothesis was that the sciatic nerve would be at the furthest distance to the infracotyloid groove at neutral+abduction+external rotation and the femoral nerve distance to the acetabulum would also be affected by the position of the hip.

## Materials and methods

**Fig. 1:**
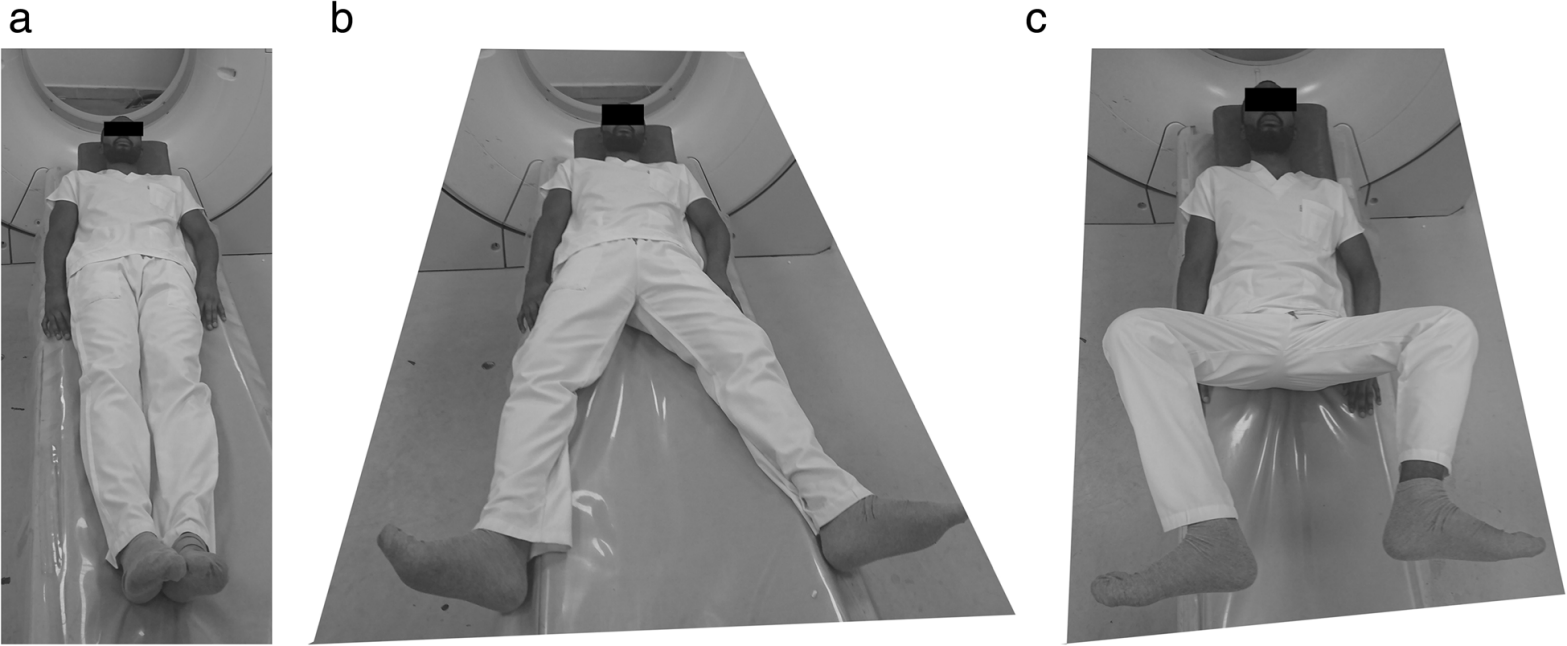
**a** Computed tomography imaging scans in neutral/supine. **b** Computed tomography imaging scans in abduction (30–45) + external rotation (20). **c** Computed tomography imaging scans in abduction (30–45) + external rotation (20) + abduction (30–45)

**Fig. 2:**
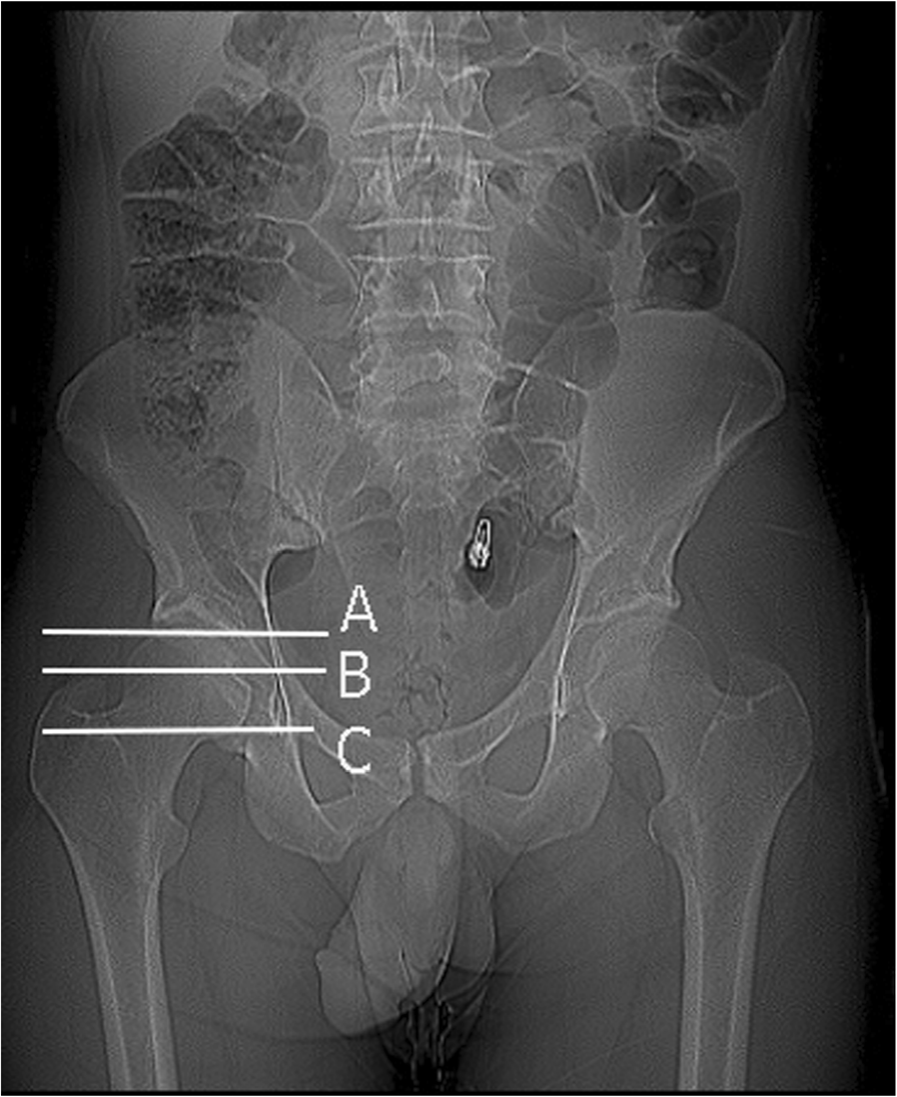
Diagram of the measurement planes on pelvic radiographs

**Fig. 3:**
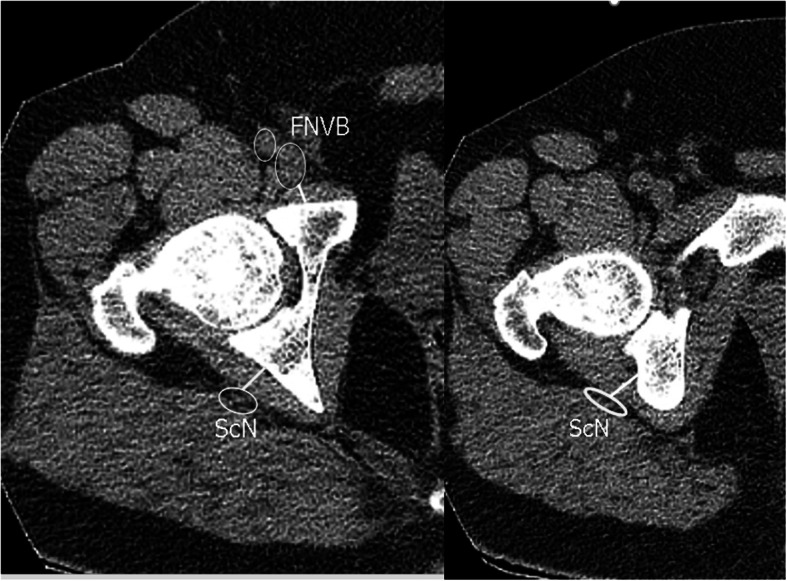
Schematic measurement of the femoral nerve and sciatic nerve at levels 1 and 2

**Fig. 4:**
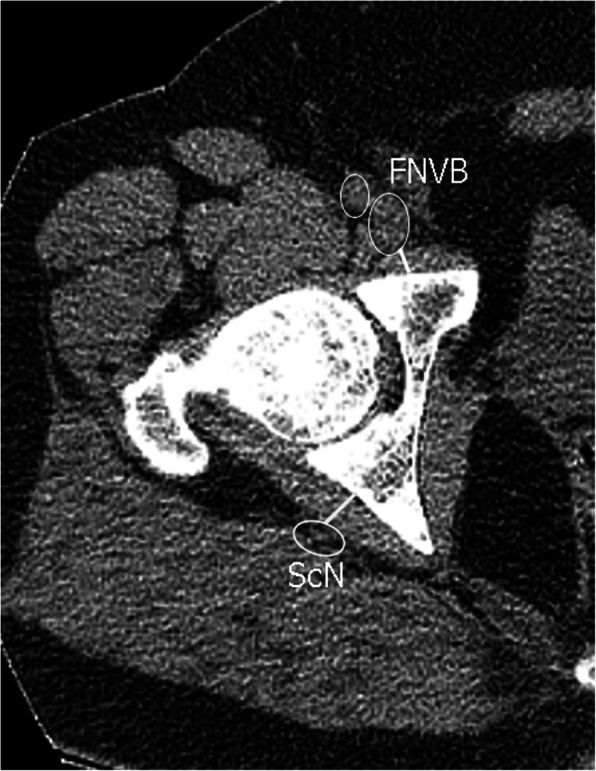
Schematic measurement of the sciatic nerve at level 3

This study included 15 healthy male adults (30 hips). The mean age is 37.3, while body mass index is 29.3 kg/m^2^. The participants were devoid of any hip or lumber spine symptoms. Patients were scanned according to standard departmental protocols at 120 kVp and 140 to 180 mAs depending on patient weight and/or girth. Computurized tomography scanograms were analyzed to rule out any signs of osteoarthritis or dysplasia. Scans were performed at three different hip positions measured by a goniometer (neutral “N” (Fig. 1a), flexion (30–45°) + abduction (30–45°) + external rotation (20°) “F” (Fig. 1b) and neutral+ abduction (30–45°) + external rotation (20°) (N_abext_) (Fig. 1c) at three different levels (sourcil “1,” middle of femoral head “2,” and lower border of triradiate cartilage “3” (Fig. 2). Using Sectra Workstation IDS7 V20.2.10.3376 (Sectra AB, Sweden), at the sourcil and middle of femoral head levels, the distances of the femoral nerve to the anterior acetabulum were measured (Fig. 3). At the triradiate cartilage and femoral head level, the distances of the sciatic nerve to the infracotyloid groove were measured (Figs. 3 and 4).

## Statistical analysis

The SPSS for Windows version 15.0 (SPSS Inc., Chicago, IL, USA) was used to place the data. The Kolmogorov-Smirnov test was used to test the distribution of the data. The Bonferonni-corrected Friedman test was used to compare the groups. The *p* value < 0.05 was set to be statistically significant.

## Results

At the sourcil level, the femoral nerve was found to be the farthest distance from the anterior acetabulum in neutral position compared to flexion or neutral plus abduction, external rotation (*p* < 0.001), while no difference was seen between the F and N_abex_ groups. The femoral head level (level 2) did not yield any differences between the different positions (*p* > 0.05) (Table 1).

**Table 1:** Mean distance between different structures

Distances (mm)			Mean ± SD[CI] mm
Femoral nerve	Sourcil level (1)	N	13 ± 1[12-13]
		F	10 ± 1[9-10]
		N_abext_	10 ± 2[9-11]
	Femoral head level (2)	N	8 ± 1[8-9]
		F	8 ± 1[8-9]
		N_abext_	8 ± 1[8-9]
Sciatic nerve	Femoral head level (2)	N	8 ± 1[8-9]
		F	10 ± 1[9-10]
		N_abext_	9 ± 1[8-9]
	Triradiate cartilage level (3)	N	11 ± 2[10-12]
		F	9 ± 2[8-10]
		N_abext_	10 ± 1[10-11]

For the sciatic nerve, at level 2, hip flexion resulted in a greater distance than in the neutral position (*p* = 0.001). Although it was still higher than that of neutral+abduction+external rotation, it did not reach statistical significance (*p* = 0.03) (Table 1).

For level 3, contrary to level 2, hip flexion caused a decrease in the distance of the sciatic nerve to the acetabulum compared to both neutral positions (N or N_abex_) (*p* = 0.001) while no difference between the two neutral positions was noted.

## Discussion

The femoral and sciatic nerves are the two main nerves of the lower extremity which could be damaged during various stages of periacetabular osteotomy [2]. Among the intraoperative variables, the position of the hip is one of the most studied ways that could be effective in preventing nerve injuries. The first “ischial” cut is one of the most demanding steps because it has to be executed without direct visual control and it is quite close to the sciatic nerve in an adult hip. Pioneers of this surgical procedure advocate extension of the hip during this cut to bring the sciatic nerve more laterally [2, 8]. However, one recent MR study reported the greatest distances of the sciatic nerve to infracotyloid groove at hip flexion/abduction/external rotation (20 mm) compared to neutral (14.8 mm) and flexion (11.8 mm) groups in healthy children. They failed to show this movement of the nerve in flexion/abduction/external rotation in two cases and explained that this was because of limited mobility of the nerve due to the lack of perineural fat tissue in slim children [5]. The present study failed to show this increase in distance with hip flexion, abduction, and external rotation. Possible explanations are that the present study included adult male participants with limited nerve mobility or that the utilization of CT opposed to MR might have caused differences in measurements “especially taking reference of bony landmarks.” However, interestingly, isolated or pure flexion increased the distance of the nerve to the bony acetabulum at the center of the femoral head.

A cadaver study reported that optimal relaxation of the nerve occurred after a full extension of the hip. Abducting the hip resulted in some relaxation while rotation had no impact [4]. The present study failed to show the effect of abduction+external rotation on the distance of the nerve to the bone.

For the femoral nerve, there does not seem to be any cadaveric or imaging study pointing out the relationship of nerve to various bony landmarks at different hip positions. One CT study advocates putting retractors (during total hip arthroplasty) as high as possible because the femoral neurovascular bundle gets closer to the acetabular anterior wall inferiorly [6]. One cadaver study reported an increase in the length (stretch) of the femoral nerve at hip extension compared to flexion or abduction or external rotation [7]. Supporting this, to reduce the risk of nerve kinking at the fragment edge, flexion of the hip is recommended. In contrast to these studies, the present study found higher distances from the nerve to the bone at the neutral position compared to flexion or neutral/abduction/external rotation. This is probably due to the fact that the distance from the nerve to the bone was measured in this CT study rather than the tension of the nerve in a cadaver.

There are some limitations to this study. We only included male adult participants with healthy hips. However, developmental hip dysplasia is mostly seen in female patients and patients with developmental hip dysplasia have altered anatomy also affecting the bone to nerve relationship and/or distance [9, 10]. A second CT was used to measure the distances, which may not be as precise as with MR for identifying soft tissue but is superior to it for bone examination. Additionally, CT had been used to quantify the distances from the nerve to bony landmarks in previous studies [6, 9]. Additional fresh cadaver studies need to be done to be more confident about the course and tension of the major nerves during various hip positions.

## Conclusion

In conclusion, in the superior part of the acetabulum or during a pubic cut of the osteotomy, the femoral nerve moves closer to the anterior acetabulum wall with hip flexion or abduction plus external rotation. In the inferior part of the acetabulum or during an ischial cut, the sciatic nerve gets closer to the ischium with hip flexion while it moves away from the bone in the middle of the acetabulum (the center of the femoral head) or during a retroacetabular cut.

## Data Availability

The datasets used and/or analyzed during the current study are available from the corresponding author on reasonable request.

## Abbreviations

N: Neutral
N_abext_: Neutral+abduction+external rotation
F: Flexion+abduction+external rotation
PAO: Periacetabular osteotomy
MR: Magnetic resonance
CT: Computed tomography
